# Gaze data reveal distinct choice processes underlying model-based and model-free reinforcement learning

**DOI:** 10.1038/ncomms12438

**Published:** 2016-08-11

**Authors:** Arkady Konovalov, Ian Krajbich

**Affiliations:** 1 Department of Economics, The Ohio State University, 1945 North High Street, 410 Arps Hall, Columbus, Ohio 43210, USA; 2 Department of Psychology, The Ohio State University, 1827 Neil Avenue, 200E Lazenby Hall, Columbus, Ohio 43210, USA

## Abstract

Organisms appear to learn and make decisions using different strategies known as model-free and model-based learning; the former is mere reinforcement of previously rewarded actions and the latter is a forward-looking strategy that involves evaluation of action-state transition probabilities. Prior work has used neural data to argue that both model-based and model-free learners implement a value comparison process at trial onset, but model-based learners assign more weight to forward-looking computations. Here using eye-tracking, we report evidence for a different interpretation of prior results: model-based subjects make their choices prior to trial onset. In contrast, model-free subjects tend to ignore model-based aspects of the task and instead seem to treat the decision problem as a simple comparison process between two differentially valued items, consistent with previous work on sequential-sampling models of decision making. These findings illustrate a problem with assuming that experimental subjects make their decisions at the same prescribed time.

## Abstract (web summary)

Learning occurs when previously rewarded actions are reinforced or when predictions are made about future consequences. Here Konovalov and Krajbich show that people who learn through reinforcement treat decisions as a comparison while those who learn by making predictions make their choices before deciding.

## Graphical abstract

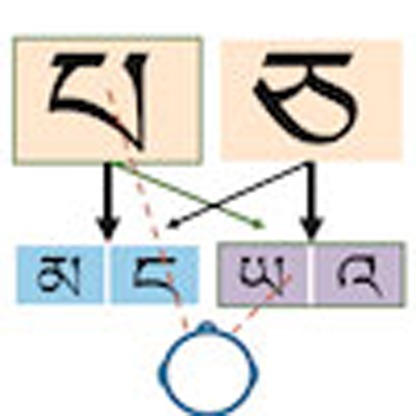

It is becoming clear that there are multiple modes of learning and decision-making. For instance, when learning which sequence of actions to choose, some decision-makers behave as if they are ‘model-free', simply repeating actions that previously yielded rewards, while others behave as if they are ‘model-based', additionally taking into account whether those outcomes were likely or unlikely given their actions^1^,^2^,^3^,^4^,^5^,^6^. Model-free behaviour is thought to reflect reinforcement learning^7^, guided by reward prediction errors^8^,^9^,^10^,^11^,^12^. On the other hand, model-based behaviour is thought to reflect consideration of the task structure, that is, decision-makers form a ‘model' of the environment. Model-based choice has been linked to goal-based behaviour^13^,^14^, cognitive control^15^, slower habit formation^16^, declarative memory^17^ and extraversion^18^. What remains unclear is whether model-free behaviour is a distinct approach to decision-making or whether it simply reflects suboptimal inference.

Behavioural and neural evidence seem to support a hybrid model where individuals exhibit mixtures of both model-based and model-free behaviour^4^,^5^,^19^. These models assume that the brain uses reward outcomes to update the expected values (‘*Q*-values') of the alternatives and then compares those *Q*-values at the time of choice. There is an implicit assumption in these models that the evaluation and choice stages occur at the same time for the model-based and model-free components, using a similar reinforcement process, but with the model-based component incorporating action-state transition probabilities.

In support of this view, recent neural evidence suggests that the brain employs an arbitration system that compares the reliability of the two systems and adjusts the relative contribution of the model-based component at the time of choice^20^. Other evidence argues for a cooperative architecture between the model-based and model-free systems^21^. Finally, model-based choices have been linked to prospective neural signals reflecting the desired state, supporting the hypothesis of goal-based evaluation^22^.

At the same time, a parallel literature has used eye tracking and sequential-sampling models to better understand value-based decision-making^23^,^24^,^25^,^26^,^27^,^28^,^29^. This work has shown that evidence accumulation and comparison drives choices and that the process depends on overt attention. Krajbich *et al.*^23^ developed the attentional drift-diffusion model (aDDM) to capture this phenomenon. The idea behind this work is that gaze is an indicator of attentional allocation^30^ and that attention to an option magnifies its value relative to the other alternative(s). Subsequent studies have identified similar relationships between option values, eye movements, response times (RT) and choices^24^,^31^. Despite this work and the vast literature on oculomotor control and visual search, the connection between selective attention and reinforcement learning, particularly model-based learning, remains unclear^26^,^32^,^33^,^34^,^35^. Some recent evidence does suggest a link between attention and choice in a simple reinforcement-learning task^26^, but in general the use of eye-tracking in this literature is just beginning^35^,^36^,^37^.

Here we seek to investigate whether model-based and model-free behaviour reflect a common choice mechanism that utilizes the same information uptake and value-comparison process (but with varying degrees of accuracy), or whether these choice modes rely on distinct processes. To do so, we use eye-tracking to study human subjects in a two-stage learning task designed by Daw *et al.*^3^ to distinguish between model-free and model-based learning. Gaze data allow us to test whether model-free and model-based subjects engage in the same choice process, and whether model-free subjects ignore task-relevant information or simply misinterpret that information. Interestingly, we find that the choices of model-free subjects show clear signs of an aDDM-like comparison process, whereas model-based subjects appear to already know which option they will choose, showing signs of directed visual search. Furthermore, model-free subjects often ignore task-relevant information, suggesting that they approach the task in a different way than the model-based subjects.

## Results

### Behavioural results

We carried out an eye-tracking experiment using a two-stage decision-making task that discriminates between model-based and model-free learning^3^. Forty-three subjects completed the experiment, which consisted of two conditions with 150 trials each.

**Figure 1:**
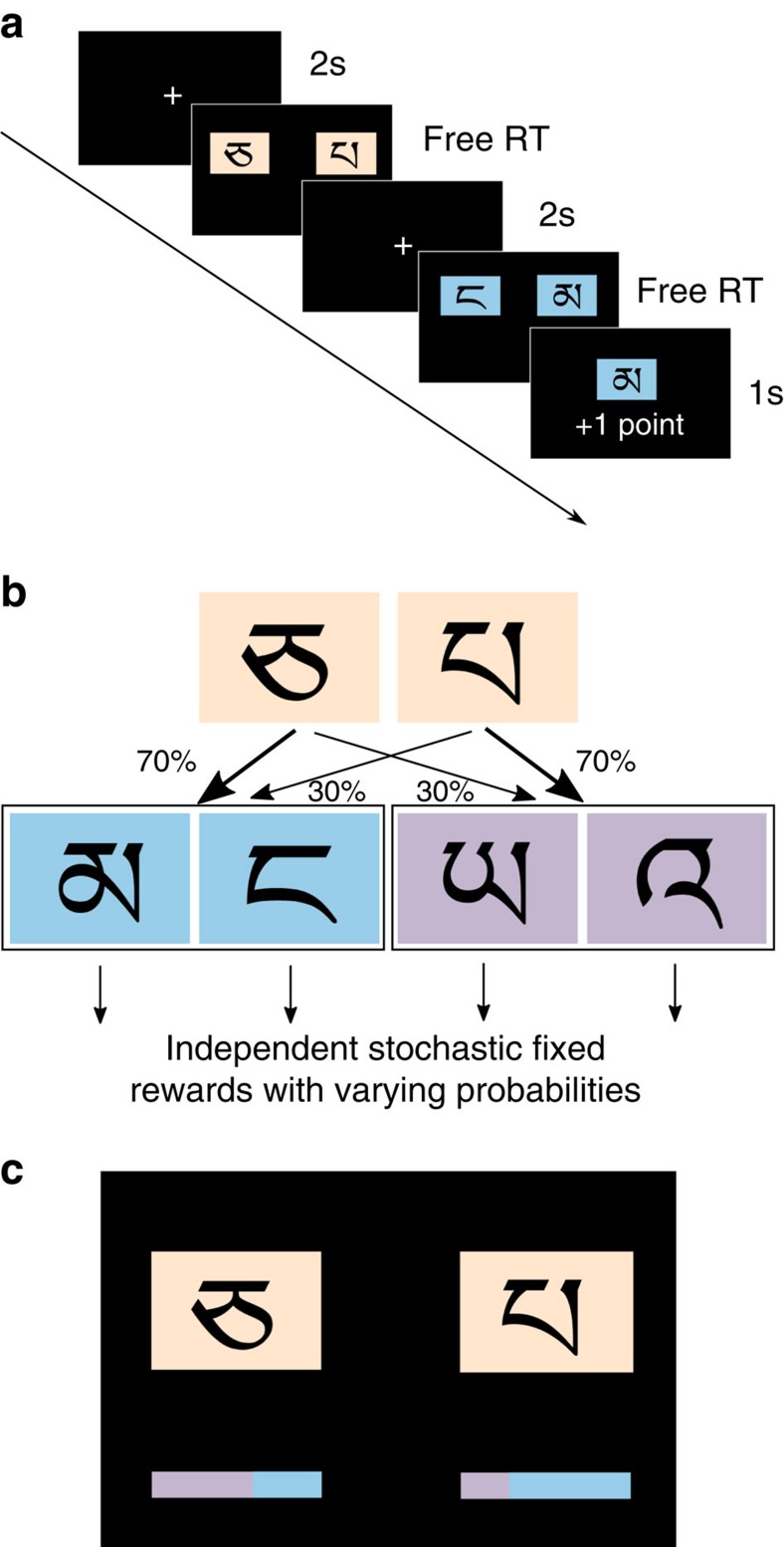
Experimental design. (**a**) Choice-trial timeline. Subjects are forced to fixate at the center of the screen for 2 s before every choice. A first stage choice between two beige symbols yields one of two second-stage states with either blue or purple symbols. Once one of the symbols is selected, it is shown in the center of the screen for 2 s, and the stochastic outcome is displayed. (**b**) Transition structure. One of the first stage symbols is more likely to lead to the blue state; the other is more likely to lead to the purple state. (**c**) First stage choice screen in the second part of the experiment. The blue/purple coloured bars indicated the change in transition probability on a particular trial (‘colour deviation').

In the first condition, we replicated the standard design. Each trial had two stages. In the first stage subjects had to make a choice between two Tibetan symbols (arbitrarily labelled ‘A' and ‘B' for further analyses) that could lead to one of two second-stage states, ‘purple' and ‘blue' (Fig. 1a). The transition was stochastic: one symbol was more likely to lead to the blue state, and the other one was more likely to lead to the purple state. Thus each first-stage symbol had a ‘common' state (probability 0.7) and a ‘rare' state (probability 0.3) associated with it. Once one of the states was reached, subjects had another choice between two symbols of the respective colour (Fig. 1b). Each of these four symbols was rewarded with a different probability that slowly drifted over the course of the experiment, independently for each symbol and irrespective of the subjects' choices.

In this task, a pure model-free learner typically repeats their first-stage choice if it led to a reward in the previous trial, irrespective of the state reached in that trial. This behaviour can be captured with a temporal-difference model that updates action values with a reward prediction error^7^. A pure model-based learner, on the other hand, typically repeats a rewarded action only if there was a common transition. We model this strategy in the standard way, using a forward-looking computational model that involves evaluation of prospective states' values using the empirically estimated transition probabilities^38^. To fit the data, we adapted a hybrid model that combines both model-free and model-based learning, implying a weight *w* between model-based and model-free action values for the first-stage symbols^3^,^14^,^39^ (*w*=1 for pure model-based and *w*=0 for pure model-free; see Methods).

The model-fitting procedure uses each subject's history of choices, transitions and rewards to estimate five free parameters. The resulting parameter distributions were in line with previous findings^3^,^22^ (Supplementary Table 1). With these parameters, the model assigns so-called ‘*Q*-values' to each option on a trial-by-trial basis. As usual, we assume that these *Q*-values are the basis for subjects' choices. In all further analyses, we refer to these hybrid *Q*-values as simply ‘*Q*-values'.

**Figure 2:**
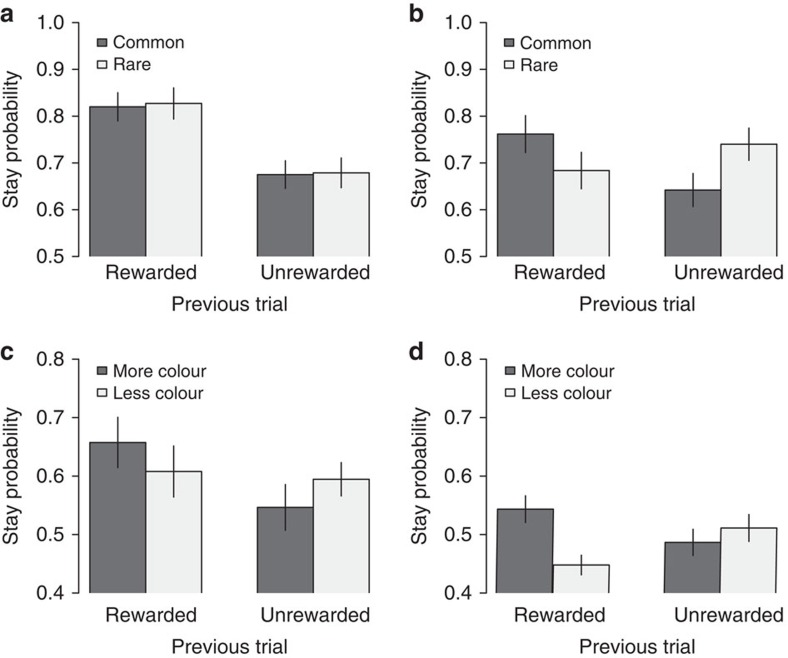
Behavioural results. (**a**,**b**) Condition 1, first stage: Probability to stay on the same symbol conditional on the previous trial's reward and the type of transition: (**a**) Twenty-two subjects that were classified as model-free learners are more likely to stay on the same symbol if it was previously rewarded. (**b**) Twenty-one subjects that were classified as model-based learners are more likely to stay only when the previous trial was rewarded and the transition was common. (**c**,**d**) Condition 2, first stage: Probability to stay on the same symbol conditional on the difference between the common state colour in that symbol's colour bar versus the other symbol's colour bar (more versus less colour) for (**c**) model-free learners and (**d**) model-based learners. Both groups are affected by the colour bar information, but model-free subjects are more likely to stay on the same symbol (60.4% versus 49.8%). Bars denote s.e., clustered by subject.

Subjects exhibited varying degrees of model-based behaviour, with the value of *w* ranging from 0 to 1. To illustrate the differences between model-based and model-free behaviour, we split our subjects into two groups based on the median *w* (0.3). In all further analyses, we use the labels ‘model-free' and ‘model-based' for the two groups defined by this median split. Consistent with prior findings we observed that the more model-free learners tended to merely repeat previously rewarded choices (mixed-effects regression, *N*=22, *P*=10^−5^, Fig. 2a, Supplementary Table 2), while the more model-based learners tended to only do so after a common transition (mixed-effects regression, *N*=21, *P*=0.005, Fig. 2b, Supplementary Table 2). Note that these results simply confirm that the model parameter *w* is indeed capturing model-based behaviour.

### Distinct gaze patterns between types

Across multiple prior experiments, the aDDM value comparison process has been linked to several patterns in subjects' gaze data^23^,^24^,^26^,^31^. While the model predicts some of them (for example, subjects tend to choose items that they are currently looking at and items that they have looked at longer over the course of the trial), other patterns are merely associated with the process (for example, first gaze location and dwell time (referred to in previous aDDM work as ‘first fixation location' and ‘fixation duration') are uncorrelated with value). Here we sought to test whether these patterns were present in the behaviour of our model-free and model-based subjects.

**Figure 3:**
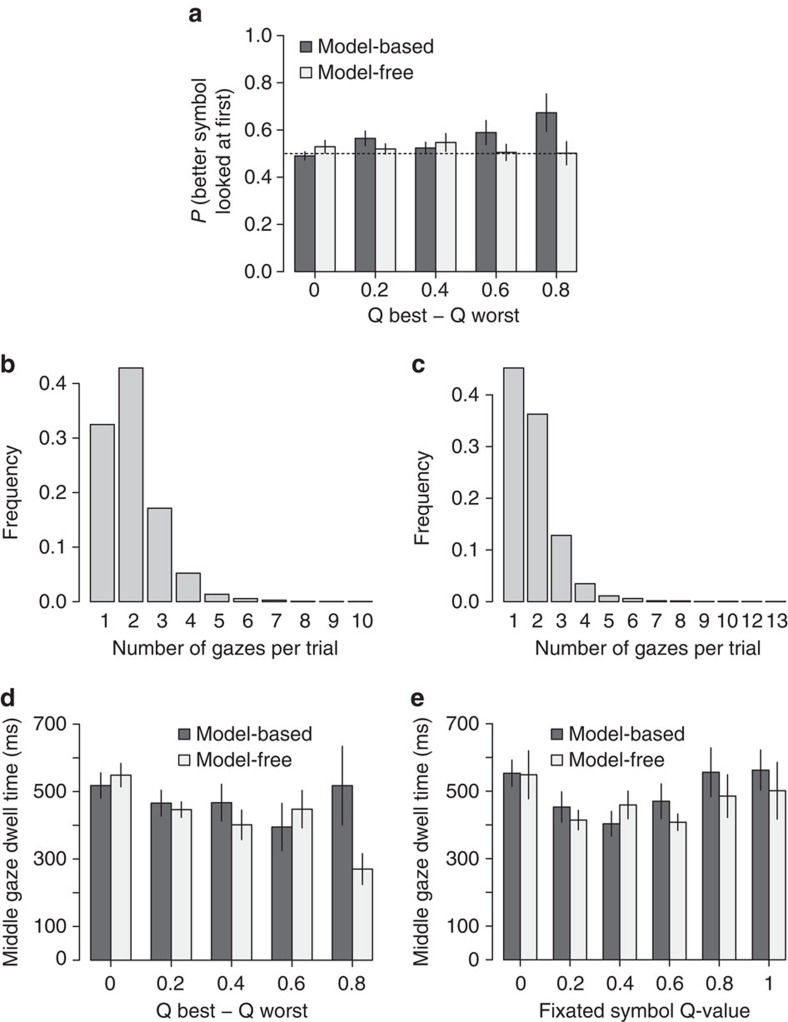
Gaze properties. (**a**) Probability that the first gaze is on the best symbol as a function of *Q*-value difference. For model-based learners, it is increasing in difference between *Q*-values and significantly different from 0.5. Bars denote s.e., clustered by subject. The dotted line represents the 0.5 probability level. (**b**,**c**) The empirical distribution of number of gazes per trial, for model-free (**b**) and model-based (**c**) learners. The distributions are significantly different. (**d**) For model-free subjects, dwell time during the trial is significantly decreasing with easier choices. (**e**) Middle-gaze dwell time is independent of the *Q*-value of the looked-at item for both. For display purposes, bins with fewer than five subjects per group were excluded from the plots. *Q*-value differences were normalized to 1 on the group level.

The initial gaze location in other experiments, both with learned values^26^ and idiosyncratic preferences^23^ has been found to be unaffected by the values of the choice options. Using a mixed-effects logit regression of initial gaze location on the higher *Q*-value item as a function of the *Q*-value difference in the first-stage decisions, we found no effects for the model-free subjects (mixed-effects regression estimates: *N*=22, intercept=0.007, *P*=0.9; absolute *Q*-value difference=0.16, *P*=0.39, no significant difference from 0.5: *N*=22, Mann–Whitney test, *W*=297, *P*=0.16, Fig. 3a), but effects significantly different from 0.5 (*N*=21, Mann–Whitney test, *W*=368, *P*=0.01) and increasing in the difference of *Q*-values, for model-based learners (mixed-effects regression estimates: *N*=21, intercept=−0.01, *P*=0.85; absolute *Q*-value difference=0.65, *P*=0.04, Fig. 3a); the difference between the *Q*-value effects for two groups was also significant (*N*=43, mixed-effects regression group dummy coefficient, *P*=0.03; regressions that include *w*'s presented in Supplementary Table 3). These results suggest that unlike model-free subjects, model-based subjects often knew ahead of time which symbols they would choose and used peripheral vision to locate them.

Consistent with this idea, model-based subjects were also more likely to look at only one of the symbols before making their first-stage choice (45% versus 32% for model-free subjects, *N*=43, *P*=0.05, controlling for *Q*-value difference using a mixed-effects regression). As a result, the two groups had significantly different distributions of the number of gazes per trial (*χ*^2^(11)=108.9, *P*<0.001; Fig. 3b,c).

In line with previous findings^23^ and characteristic of the aDDM process, during the first-stage choices, model-free middle-gaze dwells (those that were neither first nor last, so this analysis included only trials with three or more gazes) were shorter if the choice was easier (that is, the difference in *Q*-values was larger) (Fig. 3d; mixed-effects regression estimates: *N*=22, intercept=5.35, *P*=10^−12^; absolute *Q*-value difference=−2.62, *P*=0.03), while for model-based subjects this effect was only marginal (mixed-effects regression estimate: *N*=21, intercept=5.14, *P*=10^−13^; absolute *Q*-value difference=−1.79, *P*=0.07), although the difference in the coefficients between the two groups was not significant. This correlation between dwell time and choice difficulty has been noted in prior aDDM experiments and is likely due to the fact that long gazes in easier trials are more likely to result in a boundary crossing, terminating the decision.

Also consistent with previous aDDM findings, for both groups, in the first-stage choices, middle-gaze dwell time was independent of the *Q*-value for the looked-at symbol (Fig. 3e; mixed-effects regression estimates; *N*=22 and 21, *P*=0.31 for model-free and *P*=0.71 for model-based). This finding is important for thinking about causality, as it suggests that overt attention is not drawn to high-value stimuli.

At the same time, we did not find any evidence that median dwell times (two-sided *t*-test, *t*(39)=0.85, *P*=0.4) or RTs (two-sided *t*-test, *t*(41)=−0.26, *P*=0.8) were different across the two groups or varied with *w* (dwell time: Pearson *r*(41)=0.2, *P*=0.2; RTs: Pearson *r*(41)=0.14, *P*=0.39). Both groups exhibited similar-sized effects of log(RT) decreasing with the absolute *Q*-value difference (mixed-effects regression estimate; model-free subjects: *N*=22, intercept=6.84, *P*=10^−16^; absolute *Q*-value difference=−0.2, *P*=0.02; model-based subjects: *N*=21, intercept=6.81, *P*=10^−16^; absolute *Q*-value difference=−0.36, *P*=0.02), but the interaction between the *Q*-value effect and model-based behaviour was not significant (*N*=43, mixed-effects group dummy regression coefficient, *P*=0.3).

In 78% of first-stage choices, there were only one or two gazes, so if the symbol that was looked at first was not chosen during that first gaze, it was less likely to be chosen afterwards. This bias was significant for both model-free and model-based learners, but was stronger for the latter. Here we estimated a mixed-effects regression for all trials where both symbols were viewed, with the first-stage choice as the dependent variable and the following variables as predictors: absolute *Q*-value difference, first-gaze location, model-based dummy and the interaction between the first-gaze location and the model-based dummy. The results showed a negative effect of the first-gaze location with a stronger effect for model-based learners (*N*=43, intercept=0.36, *P*=0.002; *Q*-value difference (B−A)=5.03, *P*=10^−16^; first-gaze location=−0.85, *P*=10^−13^; model-based group dummy=0.22, *P*=0.19; interaction between first gaze location and model-based group dummy: −0.51, *P*=0.02; note that the significant intercept does not indicate a preference for the arbitrarily chosen symbol ‘B', rather it is a consequence of the other significant effects). This is consistent with the hypothesis that a model-based learner is looking for a particular symbol and so if they look beyond the first symbol, that symbol is unlikely to be chosen.

### Model-free behaviour is driven more by dwell time

Choices in both stages were affected not only by the predicted *Q*-values, but by gaze patterns as well (Table 1).

**Figure 4:**
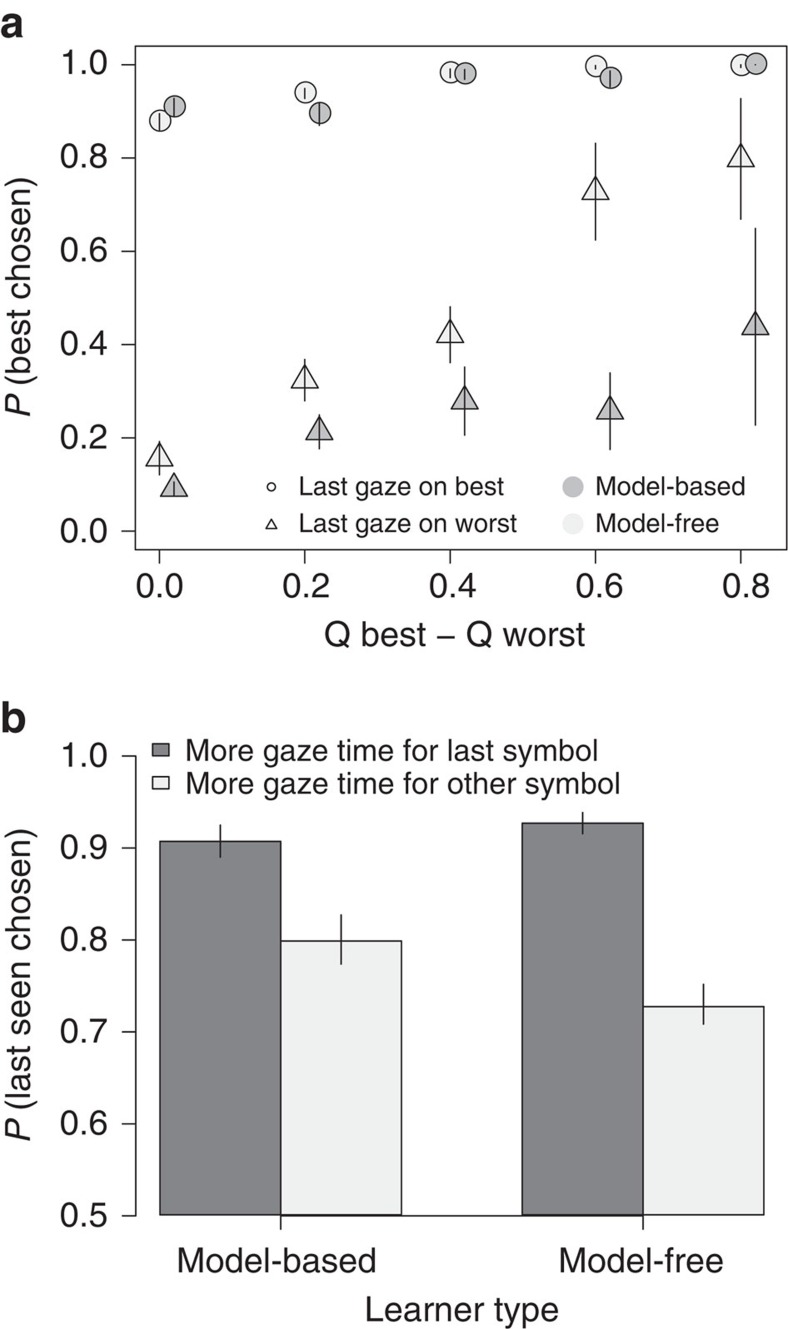
Last gaze properties. (**a**) Probability to choose the symbol with a higher *Q*-value split by the last gaze location (higher or lower *Q*-value symbol) and model-based/model-free groups. Model-based subjects tend to choose the last seen symbol irrespective of the *Q*-value difference. Bins with fewer than five subjects per group were excluded from the plots. *Q*-value differences were normalized to 1 on the group level. Bars denote s.e., clustered by subject. (**b**) Effect of gaze time advantage on probability to choose the last seen symbol. Model-free subjects are more likely to choose the last seen symbol if they spent more time looking at it during the trial.

First, we restricted out analyses to first-stage choices. Both groups' choices were affected both by the difference in *Q*-values and by the location of their final gaze (Fig. 4a). Analogous to prior work with the aDDM, when the *Q*-value difference is small, last-gaze location strongly predicts subjects' choices, whereas when the *Q*-value difference is large, attention has relatively less effect on the choice outcome and subjects overwhelmingly choose the best item irrespective of their last-gaze location. At the same time, model-based subjects' choices were more affected by the gaze location: for instance, if the last gaze was on the worse symbol (in terms of *Q*-values), they were more likely to choose that symbol, unlike the model-free subjects (two-group Mann–Whitney test, *N*=22 and 21, *W*=150, *P*=0.02).

On the other hand, model-free subjects were significantly more influenced by dwell time: they were 27% more likely to choose the last-seen symbol if the total gaze time for that symbol during the trial was longer than the gaze time for the other symbol, while this effect was only 13% in model-based subjects (Fig. 4b, two-group Mann–Whitney test, *N*=22 and 21, *W*=138, *P*=0.02). The effect of dwell time on choice is a robust effect in previous work on value-based choice, so its relative weakness in the model-based subjects again suggests that they are often using a different choice process.

To account for all the relevant factors, we used a mixed-effects logistic regression model (Table 1) to model subjects' choices. We included the following choice predictors: trial-by-trial *Q*-values generated by the computational learning model, the choice on the previous trial, last-gaze dwell time, and first and last-gaze location, as well as interactions between the gaze variables. The two groups exhibited significant differences both in the last-gaze-duration effect (Table 1, columns 1 and 3, last gaze duration coefficients) and the last-gaze-location effect (Table 1, columns 1 and 3, last gaze on B coefficients). We also observed a significant positive effect of the interaction between gaze location and a model-based dummy (*N*=43, *P*=0.02) and a significant negative effect of the interaction between these two variables and dwell time (*N*=43, *P*=0.02). These regression results were also robust to using *w* in place of the dummy variable (Table 1, column 3), and to replacing the *Q*-values with cumulative rewards for each symbol (Supplementary Table 4).

Next, we restricted this analysis to trials with only one gaze to each symbol (Table 1, columns 2 and 4; these trials constituted 40% of all trials used for eye-tracking analysis). These are the most important trials in which to look for differences between model-based and model-free subjects, since the visual search process should not require more than two gazes while the aDDM process should generally require at least two gazes. Thus the two-gaze trials are where we are most likely to observe both processes. Further supporting our earlier findings, we found that in these trials the last-gaze location effect was significantly stronger for model-based subjects (*N*=42, *P*=0.004, Table 1, column 2), while the dwell time effect was significantly stronger for model-free subjects (*N*=42, *P*=0.007, Table 1, column 2). These results were also robust to continuous *w* specification (Table 1, column 4).

Finally, if indeed model-based subjects know ahead of time what they intend to choose, we should also expect to see a similar pattern in the second-stage choices, but weaker for rare transitions than common transitions. To test this hypothesis we repeated the same analyses for the second-stage choices, taking all trials with 2 or more gazes on the second-stage symbols, and found qualitatively similar effects (Supplementary Table 5), which were indeed weaker for rare-transition trials (Supplementary Tables 6 and 7).

Taken together, these findings indicate that the behaviour of model-free subjects is consistent with an aDDM comparison process. On the other hand, model-based learners seem to often know what they are looking for (a particular symbol leading to the desired state) and thus seem to rely more on a simple visual search process. Naturally, the correspondence between the two learning types and these two processes is imperfect, but the data suggest that subjects that are more likely to employ the model-based strategy are also more likely to engage in directed visual search. Moreover, the aDDM is mostly able to capture the choice, RT, and gaze patterns (the last gaze effect and the dwell-time effect) observed in the model-free data, but it cannot account for the shifts in the model-based data: the stronger last gaze location effect (Fig. 4a) but the weaker dwell-time effects on choice (Fig. 4b; Supplementary Note 1 and Supplementary Figs 1–4). The aDDM predicts that with a change in the model parameters these effects should go in the same direction; thus the aDDM cannot seem to capture the visual-search process employed primarily by the model-based subjects.

### Visual transition cues affect choice behaviour

Our behavioural results, consistent with previous findings, suggest that model-free subjects do not properly take the transition structure into account during their first-stage choices. What is not yet known is whether these subjects are trying to track this information (and failing) or simply ignoring this aspect of the task. To answer this question, we designed a second condition of the experiment with visual cues to convey trial-to-trial variations in the transition probabilities.

In this condition, subjects completed another 150 trials of the same task, with one important difference: the transition probabilities varied randomly and independently across trials. Each trial, the probability of the common transition varied uniformly from 0.4 to 1. Mathematically the mean objective probability of the common transition was 0.7 in both conditions, but in the second condition, subjects had to update the average transition probabilities with the trial-to-trial changes in those probabilities. Here we provided subjects with on-screen visual cues indicating the deviations of the transition probabilities from their means (Fig. 1c). For simplicity we refer to the deviation of a symbol's common colour as its ‘colour deviation'. We conveyed this information with two horizontal bars (one for each symbol). Each bar was coloured partly blue and partly purple. For example, for a symbol that on average leads to the blue state with *P*=0.7, a half-blue and half-purple bar would indicate that on this trial the probability of reaching the blue state is *P*=0.7 (colour deviation=0), while a full-purple bar would indicate that on this trial the probability of reaching the blue state is only *P*=0.4 (colour deviation=–0.3). Thus a model-based subject looking to reach a particular state should utilize both the identities of the symbols and the bars.

Using a mixed-effects regression, we found that subjects indeed made use of the bars in their choices. Subjects were more likely to choose the same symbol as in the previous trial if that choice led to a rewarded common transition and the symbol's current colour deviation was greater than the other symbol's negative colour deviation. In other words, a symbol that typically leads to the blue state would be more likely to be chosen again if it led to a rewarded blue state in the last trial and its current bar contains more blue than the other bar (see Methods). This effect was highly significant (mixed-effects regression, *N*=43, *P*=10^−8^, Fig. 2c,d, Supplementary Table 2, triple interaction between reward, transition type and colour deviation difference). As before, we also found a weak model-free effect of pure reinforcement (mixed-effects regression, *N*=43, *P*=0.08) on the probability of repeating one's first-stage choice, however, we no longer observed a pure model-based effect of reward interacted with transition type (mixed-effects regression, *N*=43, *P*=0.57).

To better understand the change in these effects, relative to the first condition, we fit a modified hybrid-learning model that incorporated an additional weight parameter *v* for the colour deviations (see Methods). Optimally, a subject should weight the colour information equally to the baseline transition probability information (*w*=*v*). Instead we found that subjects heavily overweighed the colour deviations (*v*=0.67) relative to the baseline probabilities (*w*=0.16) and the model-free information (1-*v*-*w*=0.17; see Supplementary Table 1). This helps to explain the greatly diminished effects of reward and the reward*transition type interaction on subjects' choices.

Additionally, we observed that, across subjects, the model-free regression coefficient in this second condition of the experiment was negatively correlated with both *v* (Pearson *r*(41)=−0.5, *P*=0.001) from the second condition and the model-based weight *w* (Pearson *r*(41)=−0.39, *P*=0.01) from the first condition.

We can also ask whether introducing the transition information encouraged subjects to adopt a more model-based strategy. Indeed, we observed a decrease in the average model-free behaviour (1-*w* in the first condition and 1-*w*-*v* in the second condition) from 0.6 to 0.2 (*N*=43, Mann–Whitney test, *W*=350, *P*=10^−6^). While we cannot say whether the break between conditions or the additional instructions were partly responsible for this effect, we can rule out a simple effect of decreasing model-free behaviour over time. We fit a model that used two different *w*'s for the first and the second halves of the first condition, and actually observed a slight increase in the model-free weight (*N*=43, Mann–Whitney test, *W*=798, *P*=0.08).

### Model-based learners look more at the transition cues

We hypothesized that model-free subjects might pay less attention to the coloured bars, indicating that they do not make full use of the task structure. To test this hypothesis, we compared subjects' gaze patterns with their choice behaviour in both conditions of the experiment.

To measure subjects' model-based attention, we calculated the total share of dwell time on the bars compared to the symbols, as well as the probabilities of first and last gaze to the bars. All three variables were strongly correlated (Pearson *r*(41)>0.9, *P*<0.001), so we used gaze share in all further analyses.

Similar to our previous analyses, we measured the effect of the bars on subjects' choices in two ways, one with a regression model and one with the hybrid-learning model. For the first analysis, we estimated a mixed-effects logit regression predicting subjects' choices in response to the pure reinforcement effect (reward coefficient, Supplementary Table 2) the model-based effect (interaction between reward and transition) and the effect of the bar information (interaction between reward, transition and colour deviation). For the second analysis, we included the parameter *v* that captures the weight that subjects put on the colour deviations in their choices.

**Figure 5:**
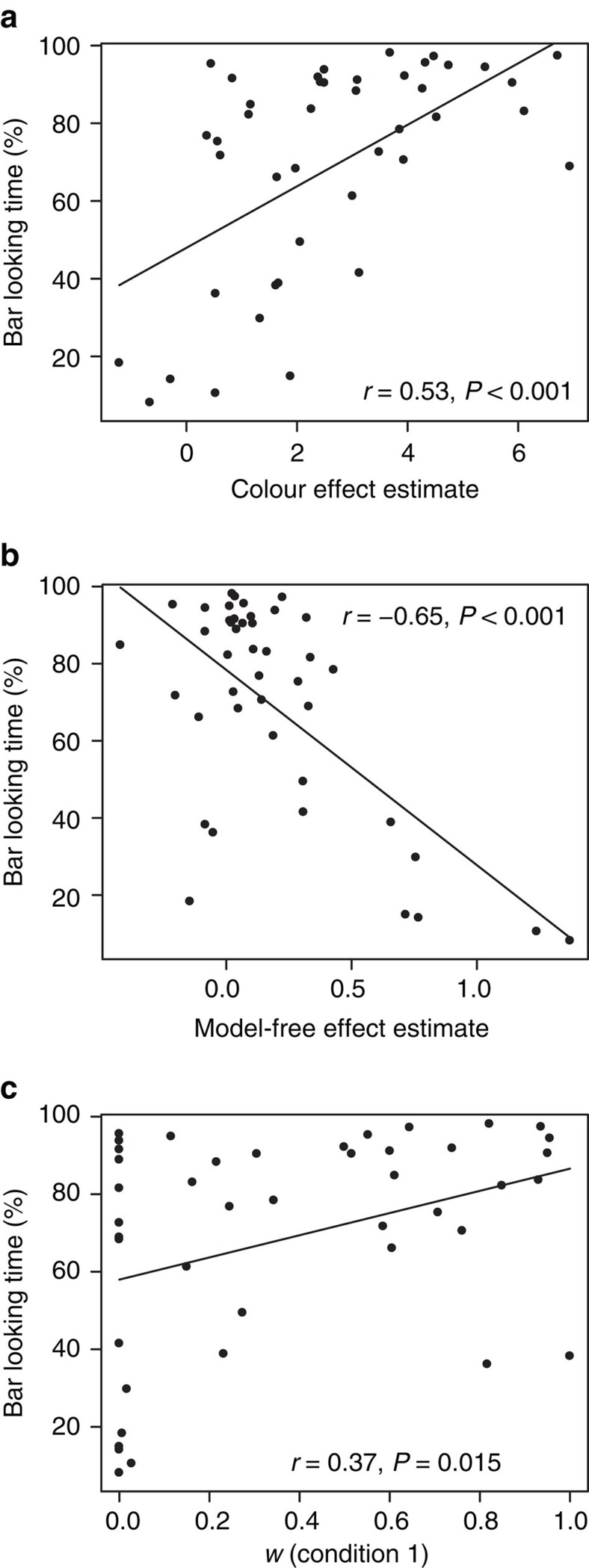
Second condition results. Across-subject correlations; lines depict linear least squares fits. Pearson correlations and *P*-values are displayed. (**a**) Correlation between the effect of the common state colour bar and the share of time spent looking at the bars in the second condition. (**b**) Correlation between the effect of reward in the second condition and the share of time spent looking at the bars. (**c**) Correlation between the model-based weight *w* in the first condition and the share of time spent looking at the bars in the second condition.

We found that the bar gaze share was positively correlated, across subjects, with both the bar-colour choice effect in the mixed-effects regression (Pearson *r*(41)=0.53, *P*=10^−5^; Fig. 5a) and the model parameter *v* (Pearson *r*(41)=0.7, *P*=10^−7^). The gaze share was also negatively correlated with the model-free (reward) coefficient in the mixed-effects model (Pearson *r*(41)=−0.65, *P*=10^−6^; Fig. 5b) and the model-free weight 1-*w*-*v* (Pearson *r*(41)=−0.7, *P*=10^−7^) of the hybrid-learning model, indicating that model-free subjects were considerably less likely to look at the visual cues.

Finally, this same bar-gaze-share measure was positively correlated with the model-based weight *w* from the first condition of the experiment (Pearson *r*(41)=0.37, *P*=0.015; Fig. 5c): on average, subjects that were classified as model-free learners were looking at the indicator bars 57% of the time, while model-based learners were looking at these bars 75% of the time. These out-of-sample results indicate that model-free subjects ignore crucial aspects of the decision task, rather than simply misinterpreting that information.

## Discussion

These results provide new insights into the intrinsic differences between model-based and model-free learning. Gaze data revealed that model-based learners seem to know what they will choose before the options even appear, while model-free learners employ an on-the-spot value comparison process that ignores the structure of the environment. The model-based learners were more likely to look at the best option first, were most likely to look at only one option, and their choices were relatively unaffected by gaze time. On the other hand, the model-free learners mostly looked at both options, often multiple times, made choices that were strongly influenced by relative gaze time, and ignored visual cues that provided information about transition probabilities. We propose that there are two distinct processes being observed at the ‘time of choice' in these multi-stage decision tasks. One is a stimulus-driven comparison process exhibited primarily by model-free subjects and the other is a simple visual search process to find an already chosen item, more typical for model-based subjects. Our novel condition with visual cues conveying model-based information was able to significantly increase subjects' reliance on transition-probability information, suggesting that the mere presence of explicit information may encourage model-based behaviour.

Our findings highlight the need to study the dynamical properties of decisions rather than treating them as static processes. The random initial gaze location and effects of gaze time and final gaze location on model-free choice align closely with previous aDDM research on decisions between food items and consumer goods^23^,^24^,^31^ as well subsequent studies using conditioned stimuli^26^ and monetary gambles^29^. This work has demonstrated that these relationships between attention and choice are a natural consequence of a value-comparison process that is biased towards the currently attended option^23^,^40^,^41^,^42^. These studies typically find that gaze location and dwell time are independent of the values of the stimuli (at least in binary choice^24^,^43^) suggesting a causal effect of attention on choice (see also^25^,^42^,^44^). Other research has argued for the opposite direction of causality, that is, that the reward process might be able to bias attention towards more valuable stmuli^32^,^45^,^46^,^47^.

Our results also have implications for the research into the neural underpinnings of model-free and model-based behaviour. Some of these studies have shown that model-based computations, particularly state prediction error evaluations, are performed at the time of reward^48^,^49^, while others show prospective computation at the ‘time of choice'^3^,^22^,^50^,^51^. Because our findings suggest that the time of choice is systematically different across groups, this means that stimulus driven neural activity must be interpreted with some caution, as it could reflect the decision process or the post-decision search process. Future neuroimaging experiments could instead investigate activity during the inter-trial interval to see if it is possible to detect model-based planning then.

There are two prominent mechanisms for how memories might be integrated to guide decisions^52^. One is prospective integration, which involves retrieving memories at the time a response is required^22^. The other is retrospective integration, which involves learning at feedback time, before the next decision is confronted^48^,^53^,^54^. The DYNA framework suggests how such learning could occur^21^ and successor representation describes how this could be extended to multi-stage environments^55^,^56^. Our findings provide support for a retrospective mechanism, which occurs between trials. However, we depart from these established models in arguing that what occurs between trials is not only learning, but the actual choice as well. It may be possible to test this directly: for instance, one would predict that a decrease in inter-trial interval might decrease model-based behaviour as there will be less time to retrospectively learn and plan for the next trial.

Finally, the distinction between model-based and model-free learners is reminiscent of the distinction between proactive and reactive cognitive control^57^. In the dual-mechanisms-of-control framework, proactive control involves anticipatory goal-related activity, while reactive control is purely stimulus driven. Similarly, we have argued that model-based behaviour involves formulating a plan before the coming decision, while model-free behaviour is stimulus driven. However, a key difference is that in most cognitive control settings the stimuli are unpredictable, whereas in our setting the same two options are present in every trial. This suggests that our results may possibly extend to more complex settings where the options vary from trial to trial^22^.

Our study also demonstrates another use for eye-tracking data in the study of decision-making: determining whether subjects make use of all of the available information. The eye-tracking results from the second condition of our experiment showed that model-free subjects do not simply misinterpret or miscalculate the task structure information, but rather tend to ignore it, even when it is presented in explicit visual form. On the other hand, model-based subjects clearly attend to this information and use it to inform their model of the task. These results corroborate previous findings in MouseLab studies of strategic bargaining^58^, where subjects who were better at forward-looking strategic thinking were also more likely to collect information about payoffs in future states. Moreover, providing these visual cues seemed to reduce the overall amount of model-free learning, suggesting a potential remedy to this suboptimal behaviour.

Further research into the dynamical properties of model-based and model-free behaviour are clearly needed to gain a better understanding of the distinction between these modes of learning. Our study has provided an initial glimpse into the different mechanisms underlying these behaviours, but more work is needed to link the eye-tracking data to neuroimaging results. We hope that this research will fuel further investigation into the ties between static models of learning and dynamical models of choice, which will certainly yield deeper insights into these core topics in decision science.

## Methods

### Subjects

Forty-five students (19 female) at The Ohio State University were recruited from the Department of Economics subject pool. Subjects were paid based on their overall performance in the decision task, at a rate of 5¢ for one reward point, with a minimum payment of $5 as a show-up fee. Subjects earned an average of $16. One subject experienced software crashes during the experiment, and another one explicitly failed to understand the task, so these two subjects' data were excluded from the analysis. The Ohio State University Internal Review Board approved the experiment, and all subjects provided written informed consent.

### Two-stage decision task

In the first condition of the experiment, subjects completed 150 trials of a two-stage Markov decision process task^3^, with two short breaks every 50 trials (Fig. 1a). On each trial, the first stage involved a choice between two Tibetan symbols that had different probabilities of transition to two possible second-stage states (blue and purple, by the colour of the boxes that contained the symbols). One symbol was more likely to lead (on average) to the blue state, while the other one was more likely to lead to the purple state (Fig. 1b). On every trial, for each first-stage symbol the transition probabilities to the common state were independently and randomly sampled from a uniform distribution in the interval between 0.4 and 1, resulting in an average of *P*=0.7 for each symbol. The other, rare state, was reached with probability 0.3. Subjects were instructed that each symbol was more likely to lead to one of the second-stage states, but they had to identify the transition probabilities on their own.

In the second stage, subjects were required to choose between two symbols in the state they reached (blue or purple). Each of the four second-stage symbols had an independent probability of yielding a fixed reward. During the course of the experiment, these probabilities drifted independently in the range from 0.25 to 0.75 according to slow Gaussian walks with mean=0 and s.d.=0.025 to facilitate learning and exploring different states.

In each stage, the position of the symbols on the screen was randomized. Choices were made using a keyboard, and every choice was followed by a white frame around the chosen symbol for 0.5 s. All choices had free RT. After the second-stage symbol was chosen, it was displayed at the center of the screen, with the outcome shown in the bottom part of the screen (either ‘+1 point' or ‘0 points').

Before starting the task, subjects were introduced to the rules of the task, including a short practice on each part of the task, and a 30-trial practice session with different stimuli.

### Two-stage decision task with visual transition cues

In the second condition of the experiment, subjects completed another 150 trials of the two-stage task with a modified first-stage decision screen. Under each symbol we displayed visual cues for the respective deviations of the transition probabilities (Fig. 1c). The cues were presented in the form of coloured (blue and purple) bars. The horizontal size of each bar was equal to the horizontal size of the symbol boxes. Each bar showed the deviation of the particular trial transition probability from the average (0.7). At the average, each bar had blue and purple segments of equal size. If the probability of transition to a common state was sampled closer to 1, the segment of that state's colour had a larger share of the bar, proportional to the absolute deviation from 0.7. On the other hand, if the transition probability approached 0.4, that segment's share was smaller. Subjects went through additional instructions and training with the bars to ensure comprehension of the task.

The second stage decision screen was exactly the same as in the first condition of the experiment (see above). Subjects were instructed that the reward probabilities for all four second-stage symbols were randomly reset for this condition, but that the first-stage symbols retained their transition probabilities.

### Eye-tracking methods

Subjects' gaze data was recorded using an EyeLink 1000+ desktop-mounted eye-tracker with a chin rest and sampled at 1000 Hz. Before every choice, subjects were required to fixate at the center of the screen for 2 s, or the software did not allow them to proceed. This ensured unbiased initial gaze positions. The task was created and displayed using Matlab and Psychtoolbox^59^. The chin rest was placed at 65 cm away from the screen, and the screen resolution was set at 1920 × 1080.

### Eye-tracking data analysis

The following procedure was applied to the gaze position data. The size of a symbol was set to 400 × 290 pixels. A gaze on a symbol was recorded if the gaze position was within a region of interest (ROI) that included the symbol itself and a 50-pixel margin, so the horizontal distance between ROIs was 460 pixels. The ROIs for the bar indicators were set at the same size as the symbol ROIs centered around the bars. Vertically, the symbols were centered at 33% of the distance from the top of the screen, and the bars were placed at 80% distance from the top of the screen.

Trials with no gaze on the ROIs were excluded from all gaze analyses (the mean number of such trials in the first stage was 20 out of 150 per subject, with 30 subjects having less than 20 trials excluded; there was no significant difference in the number of excluded trials between model-based and model-free learners, two-sided *t*-test, *N*=22 and 21, *P*=0.77). The main results were also robust to focusing solely on these 30 subjects (Supplementary Figs 5–7, Supplementary Table 8).

Gaps between two gazes on the same ROI were interpreted as a blink or a technical error (for example, eye-tracker losing the pupil) and treated as one gaze to the same item. Gaps between gazes on two different ROIs were discarded.

### First condition choice analysis

We implement a variant of a well-known hybrid learning model^38^ that assigns so-called action-state *Q*-values to every action and combines the SARSA(λ) (state-action-reward-state-action) model-free reinforcement and a forward-looking model-based strategy that makes use of the empirical transition probabilities to evaluate expected values of the first-stage choices.

The model-free learning strategy uses only reward information to update the *Q*-values. These values are initialized to zero at the beginning of the experiment. Let *a*_1_ be the symbol chosen in the first stage of the task, and *a*_2_ be the second-stage choice (subscripts generally indicate stage number). Then, after a trial *t* is completed and a reward *r*(*t*)∈{0,1} is received, the chosen second-stage symbol *a*_2_'s *Q*-value *Q*_2_ is updated in the following way:


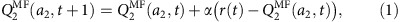


where *α* is a learning rate parameter, and *r*(*t*)−*Q*_2_^MF^(*i*,*t*) is a reward prediction error. This process is identical for both model-free and model-based decision makers as there is no stochastic transition after this stage.

The value of the symbol chosen in the first stage is also updated through the reinforcement process, using both the second-stage reward prediction error and the prediction error that comes from the difference between the obtained and expected value of the second-stage state:


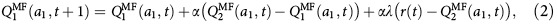


where *λ* is an eligibility trace parameter that captures the effect of the second-stage prediction error on the first-stage action value.

The model-based learning strategy incorporates the empirical transition probabilities into the updating process^38^:


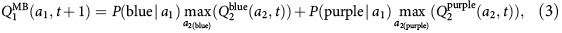


where *P*(blue|*a*_1_) and *P*(purple|*a*_1_) are the respective transition probabilities after choosing action *a*_1_ which are calculated using Beta-Binomial Bayesian updating:


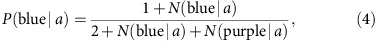


where *N*(blue|*a*) and *N*(purple|*a*) are the numbers of times the blue or purple state was reached after making a choice *a*.

The hybrid model *Q*-value for each first-stage choice is calculated using a convex combination of the model-free and model-based action values:


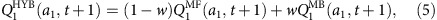


where *w* is a weight parameter restricted between 0 (pure model-free strategy) and 1 (pure model-based strategy).

A logit discrete choice model is assumed for both stage choices, with probability of the second stage choice computed as


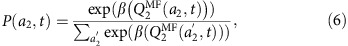


and the first stage as


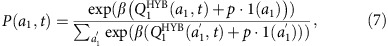


where *β* is a traditional choice ‘inverse temperature' parameter, 1(*a*_1_) is an indicator function that returns 1 if the same symbol was chosen in trial *t*−1, and 0 otherwise, and *p* is a parameter that captures ‘stickiness' in first-stage choices.

The hybrid model has 5 free parameters: *α*,*β*,*λ*,*p*,*w*. We do not use different *α*'s and *β*'s for the two stages for several reasons: (a) a larger number of parameters adds more noise to the estimation of our parameter of interest, *w*, (b) previous studies do not provide conclusive statistical evidence of a difference between first and second stage *α* and *β* values in the population^3^,^22^, and (c) we find that for almost all of the subjects (40/43) the simpler model fits the data better, in terms of the Bayesian information criterion (BIC), than the model with two additional parameters.

We fit the model individually to each subject's data using a maximum likelihood estimator and the probability formulas defined above. We restrict *α*, *λ* and *w* to lie between 0 and 1, *β* to be positive, and use a Nelder–Mead optimization procedure with 10,000 random starting points to ensure the achievement of global maxima. Obtained values were used to derive hybrid *Q*-values on a trial-by-trial basis for each subject individually.

### Second condition choice analysis

In the second condition of the experiment, choices were influenced by visual cues presented on the screen. For this condition, we used a modified model that assumed two weight parameters instead of one. For the sake of notation simplicity, let *p*_state_=*P*(state|*a*_1_) be the empirical probability of transition to a particular *state* after the first-stage action *a*_1_, estimated via the Baysesian updating formula in (4), and Δ*p*_state_=*p*(*t*)−0.7 be the deviation of the trial transition probability from the mean. Then the hybrid *Q*-value for action *a*_1_ is defined as


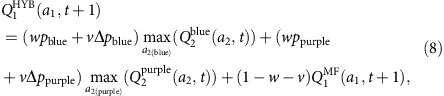


where *w* is a weight assigned to the mean transition probability that has to be inferred from the symbol's identity, and *v* is a weight assigned to the correction provided by the colour bars.

In all other aspects, the computational model in this part of the experiment is equivalent to the model in the first part.

### Regression analysis

In addition, following previous literature^3^,^18^, we ran a hierarchical logistic regression. All data was fit using the mixed-effects regression in lme4 package^60^ in *R*, and all coefficients were treated as random effects at the subject level. The dependent variable was the choice of the same first-stage symbol that was chosen in the previous trial (stay=1, switch=0).

In the first condition of the experiment, it was regressed on the previous trial reward (1 or 0) and type of the previous trial state transition: 1 if the state reached was common for the chosen symbol and 0 if it was rare, as well as the interaction of these two variables. In this regression, the coefficient on the reward reflects model-free choice, and the coefficient on the interaction reflects model-based choice: a model-based subject would repeat their choice if the transition was common, and would switch to the other symbol if the transition was rare.

In the second condition of the experiment, we used an additional continuous variable colour that was equal to the difference between the colour deviation for the common transition for the symbol chosen in the previous trial and the colour deviation for the same state for the other symbol. For example, if symbol A was chosen on the previous trial, and its common state was blue, then the colour variable in the current trial would be equal to the blue area for symbol A minus the blue area for symbol B. In this regression, we used all three regressors as well as their interactions. In addition to the effects described above, the interaction between reward, transition type and colour, measures the effect of the visual cues on choices in the first stage of the task.

### Data availability

The data that support the findings of this study are available from the corresponding author upon request.

## Additional information

**How to cite this article:** Konovalov, A. *et al.* Gaze data reveal distinct choice processes underlying model-based and model-free reinforcement learning. *Nat. Commun.* 7:12438 doi: 10.1038/ncomms12438 (2016).

## Supplementary Material

Supplementary Information Supplementary Figures 1 – 7, Supplementary Tables 1 – 8 and Supplementary Note 1

## Figures and Tables

**Table 1:** Choice predictors.

*P* (B chosen)	Two groups		Continuous *w*	
	Two or more gazes	Exactly two gazes	Two or more gazes	Exactly two gazes
Intercept	−2.49^***^	−0.06	−2.43^***^	0.01
	(0.29)	(0.44)	(0.30)	(0.45)
QB−QA	3.72^***^	3.92^***^	3.66^***^	3.89^***^
	(0.41)	(0.48)	(0.40)	(0.51)
B chosen at *t*−1	1.68^***^	1.85^***^	1.69^***^	1.85^***^
	(0.27)	(0.33)	(0.26)	(0.33)
First gaze on B	1.20^***^		1.20^***^	
	(0.21)		(0.21)	
Last gaze on B	2.06^***^	−1.21	2.00^***^	−1.21
	(0.36)	(0.72)	(0.38)	(0.76)
Last gaze dwell time	−0.39^***^	−0.84^***^	−0.38^***^	−0.83^***^
	(0.09)	(0.16)	(0.09)	(0.17)
Model-based/*w*	0.84^***^	1.58^***^	−0.75	−2.04^**^
	(0.14)	(0.27)	(0.46)	(0.76)
Last gaze time × last gaze on B	−0.50	−1.45^**^	0.83^***^	1.56^***^
	(0.35)	(0.54)	(0.14)	(0.29)
Model-based/*w* × last gaze on B	1.15^*^	2.83^**^	1.53^*^	3.54^*^
	**(0.50)**	**(0.99)**	**(0.68)**	**(1.39)**
Model-based/*w* × last gaze time	0.18	0.45*	0.19	0.55*
	(0.12)	(0.19)	(0.16)	(0.27)
Model-based/*w* × last gaze on B × last gaze time	−0.39^*^	−0.92^**^	−**0.44**^**†**^	−1.09^*^
	**(0.17)**	**(0.34)**	**(0.25)**	**(0.49)**
AIC	2027.81	1324.87	2028.99	1311.58
BIC	2267.11	1541.19	2268.30	1488.05
Log likelihood	−974.90	−624.44	−975.50	−624.79
Number of trials	3415	2192	3415	2192
Number of subjects	43	42	43	42

^***^P<0.001, ^**^P<0.01, *P<0.05, ^†^*P*<0.1. Standard errors in parentheses.

Fixed effects coefficient estimates of first-stage choice regressions in part 1 of the experiment (symbols are arbitrarily labelled A and B) using mixed-effects logistic models. Columns 1 and 2 display results that used a group dummy variable to compare model-based and model-free subjects coefficients; columns 3 and 4 present the results using a continuous value of *w*. Model-based behaviour is more affected by the gaze location, while model-free behaviour is more influenced by dwell time. The binary dependent variable was equal to 1 if symbol ‘B' was chosen; QB−QA is the hybrid *Q*-value difference estimated from the computational model; ‘B chosen at t−1', ‘Last gaze on B', and ‘First gaze on B' are binary variables; ‘Last gaze time' is duration of the last gaze on the particular trial; ‘model-based' was equal to 1 if the subject was in the model-based group (*w*<0.3). The coefficients of primary interest are shown in bold. AIC, Akaike information criterion; BIC, Bayesian information criterion.
