# Supplementary material for: Gaze data reveal distinct choice processes underlying model-based and model-free reinforcement learning
Source: Nat Commun. 2016 Aug 11;7:12438. doi: 10.1038/ncomms12438 (PMC4987535; doi:10.1038/ncomms12438)
Supplement: Supplementary Information — Supplementary Figures 1 – 7, Supplementary Tables 1 – 8 and Supplementary Note 1 [file ncomms12438-s1.pdf]

SUPPLEMENTARY MATERIALS

"Gaze Data Reveal Distinct Choice Processes Underlying Model-Based and Model-Free Reinforcement Learning"

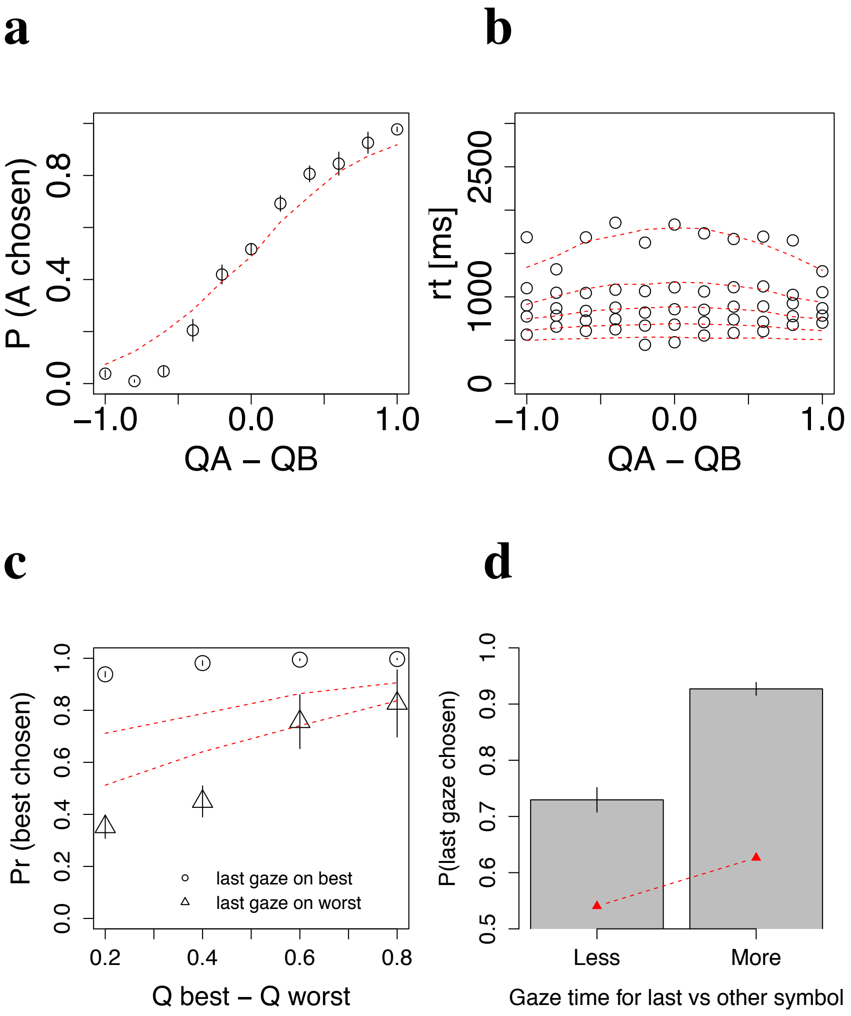

Supplementary Figure 1. The aDDM fit to model-free (MF) subjects' data as described in Supplementary Note 1. **(A)** Choice as a function of Q-value difference. **(B)** RT quintiles as a function of Q-value difference (see Ratcliff & McKoon 2008). **(C)** Replication of Fig. 4a in the paper using only the MF data. **(D)** Replication of Fig. 4b in the paper using only the MF data. Black symbols and grey bars represent the data with standard error bars, and the dotted red lines show the model fits (d = 0.0022, σ = 0.038, θ= 0.55, t_{er} = 350).

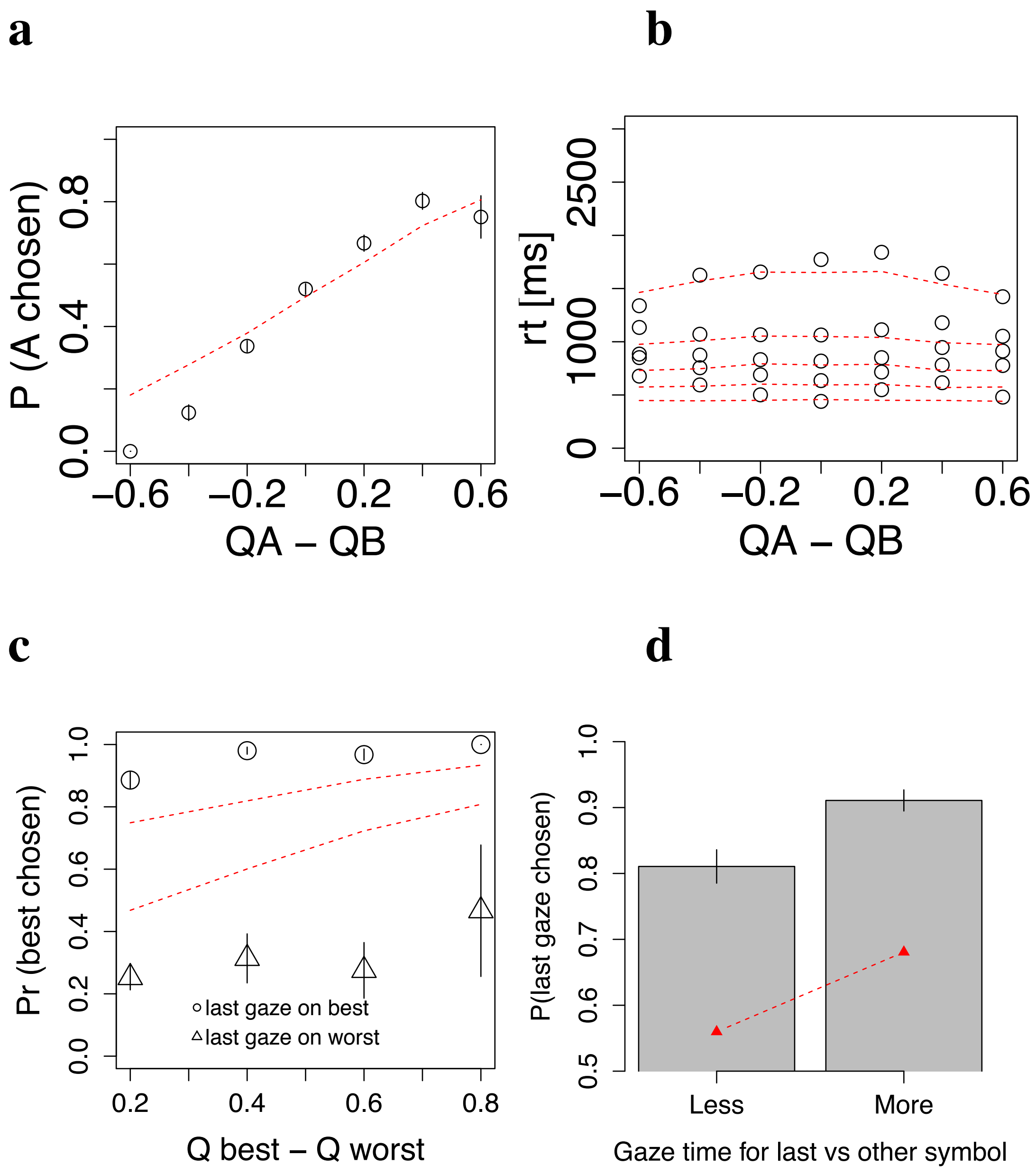

Supplementary Figure 2. The aDDM fit to model-based (MB) subjects' data as described in Supplementary Note 1. **(A)** Choice as a function of Q-value difference. **(B)** RT quintiles as a function of Q-value difference. **(C)** Replication of Fig. 4a in the paper using only the MB data. **(D)** Replication of Fig. 4b in the paper using only the MB data. Black symbols and grey bars represent the data with standard error bars, and the dotted red lines show the model fits (d = 0.0025, σ = 0.038, θ= 0.45, t_{er} = 275).

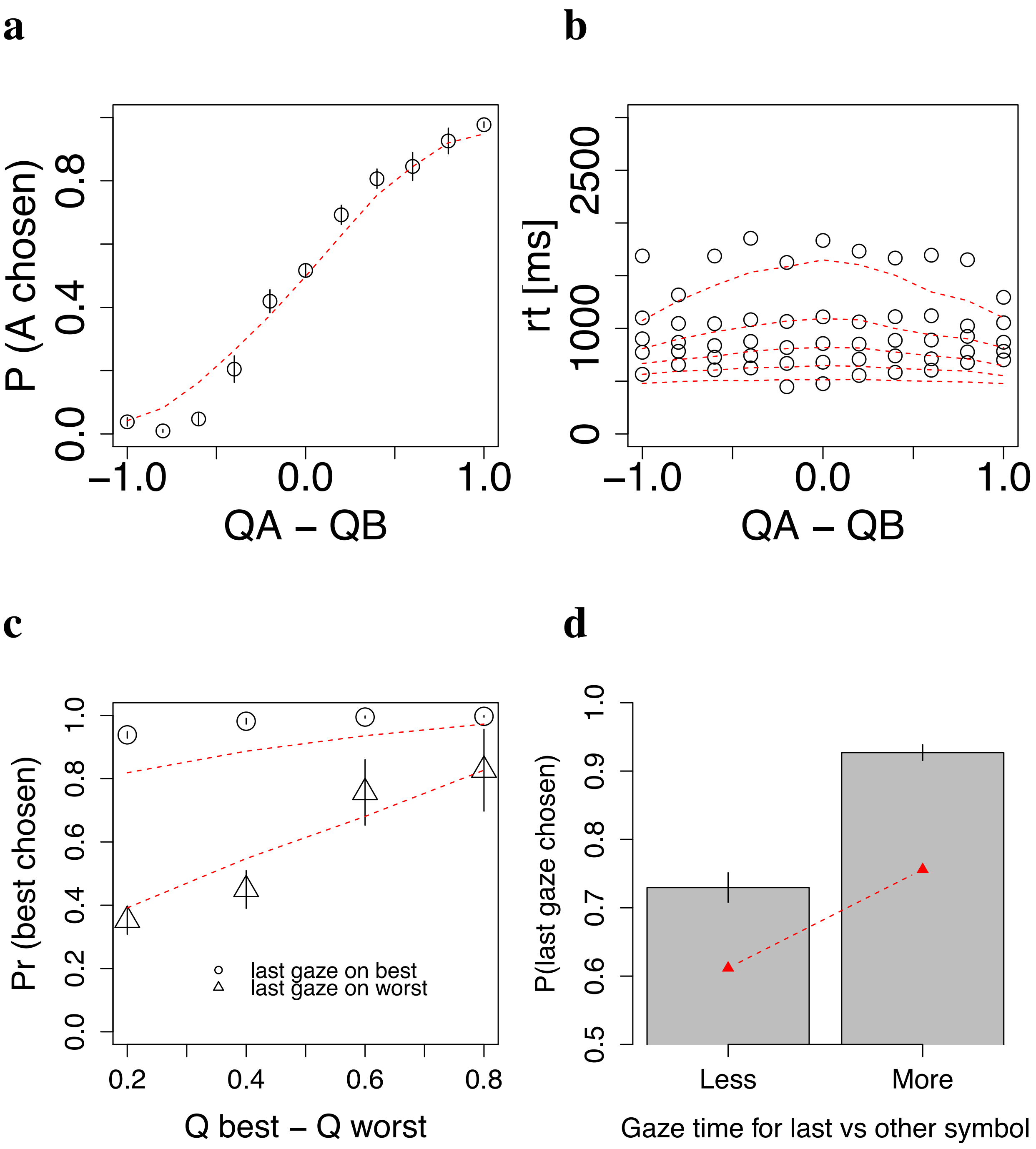

Supplementary Figure 3. The aDDM fit to MF subjects' data using $\theta = 0.3$ and adjusting the other model parameters accordingly. (A) Choice as a function of Q-value difference. (B) RT quintiles as a function of Q-value difference. (C) Replication of Fig. 4a in the paper using only the MF data. (D) Replication of Fig. 4b in the paper using only the MF data. Black symbols and grey bars represent the data with standard error bars, and the dotted red lines show the model fits (d = 0.0039, σ = 0.033, θ= 0.3, t_{er} = 350).

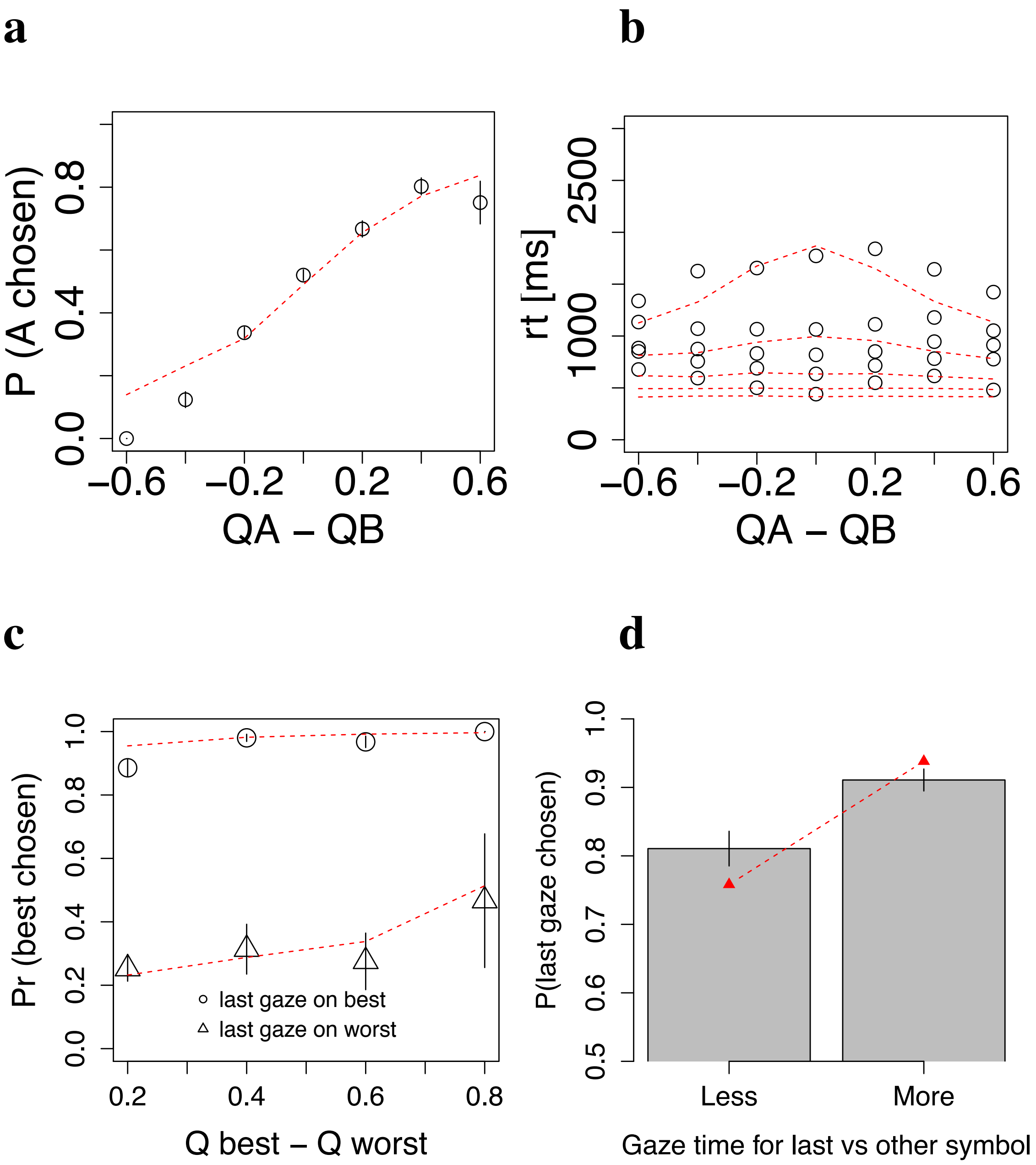

Supplementary Figure 4. The best aDDM fit to MB subjects' data from the set of parameters described in Supplementary Note 1. **(A)** Choice as a function of Q-value difference. **(B)** RT quintiles as a function of Q-value difference. **(C)** Replication of Fig. 4a in the paper using only the MB data. **(D)** Replication of Fig. 4b in the paper using only the MB data. Black symbols and grey bars represent the data with standard error bars, and the dotted red lines show the model fits (d = 0.0059, σ = 0.033, θ= 0, t_{er} = 275).

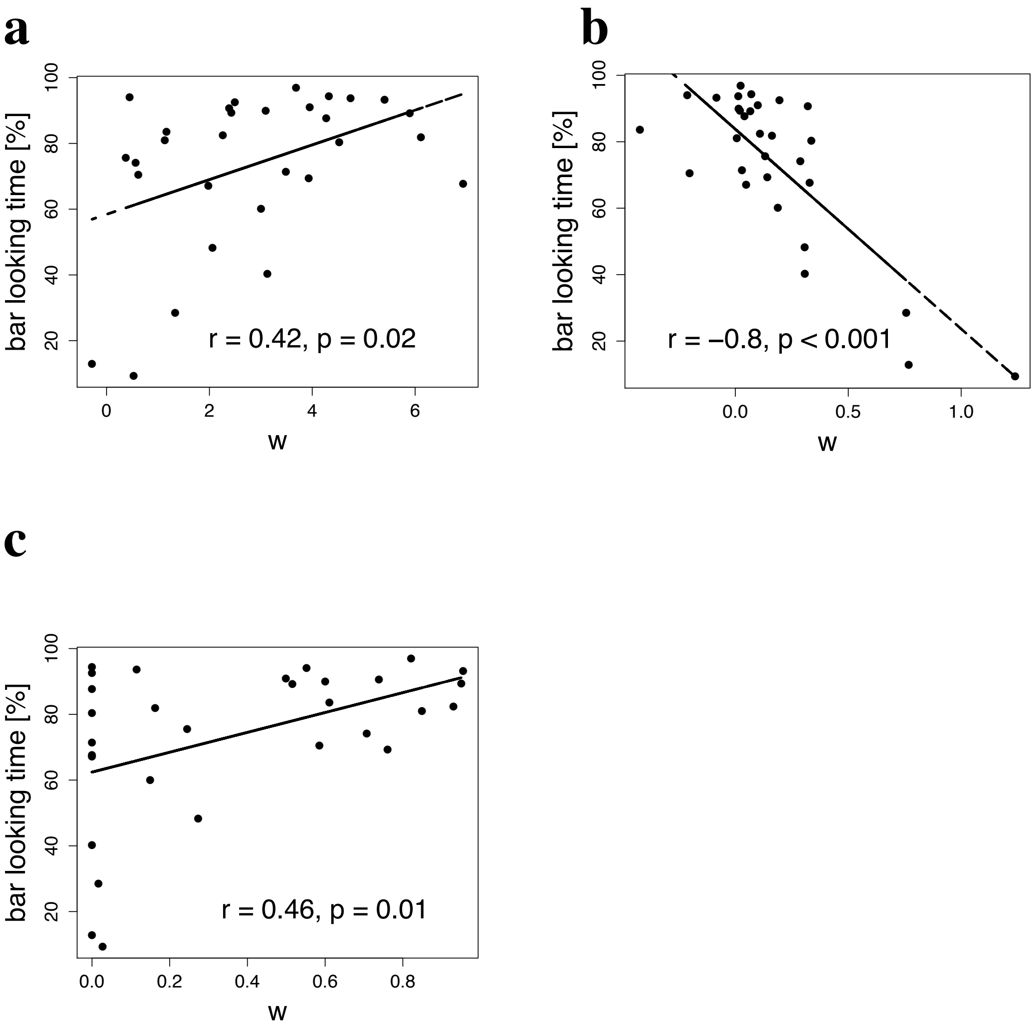

Supplementary Figure 5. Figure 5 excluding the 13 subjects whose zero-gaze-trial rate was above average.

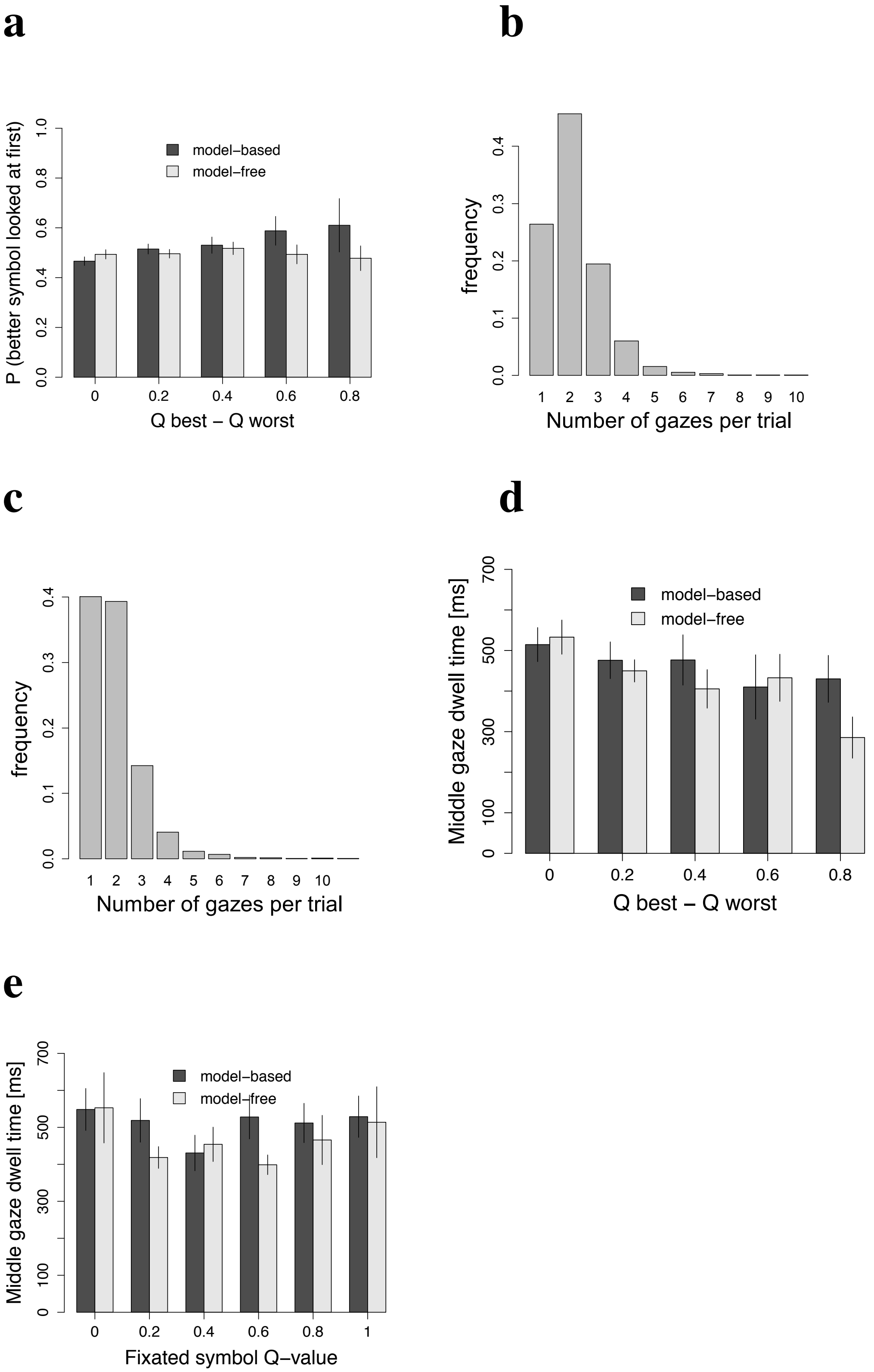

Supplementary Figure 6. Figure 3 excluding the 13 subjects whose zero-gaze-trial rate was above average.

a

b

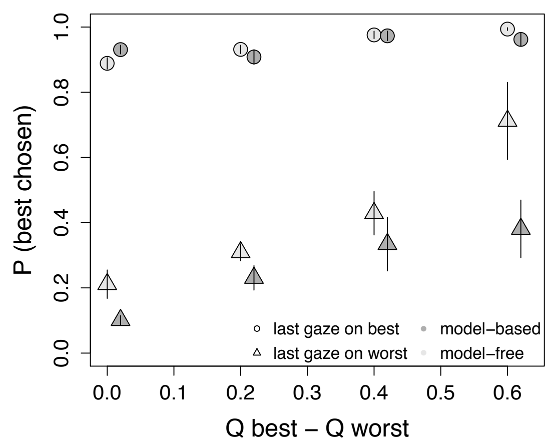

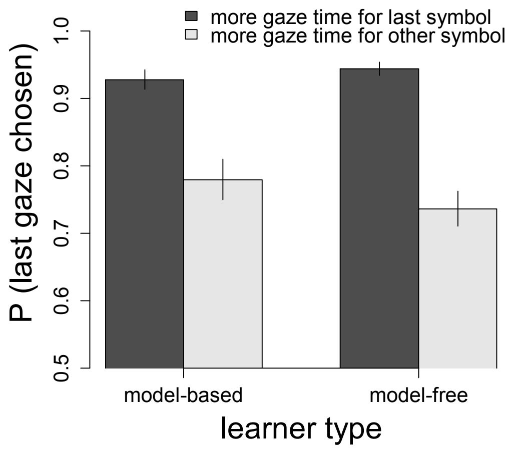

Supplementary Figure 7. Figure 4 excluding the 13 subjects whose zero-gaze-trial rate was above average.

	Condition 1		Condition 2	
	**mean**	**sd**	**mean**	**sd**
α	0.409	0.247	0.484	0.273
β	3.827	2.175	4.775	2.703
λ	0.584	0.365	0.580	0.389
p	0.254	0.255	0.129	0.452
w	0.393	0.364	0.161	0.240
v	-	-	0.670	0.403
LL	-152.312	35.031	-157.785	33.245

Supplementary Table 1. Means and standard deviations of model parameters across subjects, in condition 1 and condition 2 of the experiment. The computational hybrid learning model was fit to each subject individually using a maximum likelihood estimator. α is the learning rate, β is the inverse temperature parameter, λ is the eligibility trace parameter, p is the stickiness of choice, w is the weight of the model-based strategy (=0 for pure reinforcement and = 1 for pure model-based behavior), v is the weight on the color deviations in the second condition of the experiment. LL is the maximized posterior likelihood.

P (stay)	Part 1 all data	Part 1 model-free	Part 1 model-based	Part 2 all data
Intercept	0.96^{***}	0.74^{***}	1.15^{***}	0.20
	(0.13)	(0.15)	(0.20)	(0.11)
reward	0.41^{*}	1.13^{***}	-0.25	0.22
	(0.19)	(0.26)	(0.22)	(0.13)
transition	-0.23	0.05	-0.49^{*}	-0.06
	(0.13)	(0.15)	(0.21)	(0.08)
reward:transition	0.46^{*}	-0.16	1.01^{**}	0.07
	(0.21)	(0.22)	(0.36)	(0.12)
color				0.02
				(0.17)
reward:color				-1.46^{***}
				(0.32)
transition:color				-0.16
				(0.21)
reward:transition:color				2.75^{***}
				(0.48)
AIC	6861.84	3380.35	3478.78	8285.01
BIC	6956.55	3465.68	3563.46	8582.68
Log Likelihood	-3416.92	-1676.18	-1725.39	-4098.51
Num. obs.	6407	3278	3129	6407
Num. groups: id	43	22	21	43

$^{***}p < 0.001$, $^{**}p < 0.01$, $^{*}p < 0.05$. Standard errors in parentheses.

Supplementary Table 2. Probability of choosing the same symbol as in the previous trial (1 = stay, 0 = switch) conditional on the previous trial's reward outcome (1 = rewarded, 0 = unrewarded) and the type of transition (1 = common, 0 = rare), fixed effect at population level. All regression models are mixed effect logistic regressions performed in lme4 R package (formula: stay ~ reward*transition + (1+ reward*transition | subject)). Column 1 shows the pooled data exhibiting a mixture of pure reinforcement and model-based learning. Columns 2 and 3 show the results for model-free and model-based learners, classified using a full hybrid computational model. Column 4 shows the pooled data for the second part of the experiment (formula: stay ~ reward*transition*color + (1+ reward*transition*color | subject)). The *color* variable is the difference between the color deviation for the chosen symbol and the negative color deviation for the other symbol.

	Group dummy		Continuous w	
P (look at higher Q first)	**All data**	**Without outlier**	**All data**	**Without outlier**
Intercept	0.04	0.09	0.05	0.11
	(0.08)	(0.07)	(0.08)	(0.07)
abs(QB - QA)	0.03	-0.07	0.01	-0.09
	(0.18)	(0.16)	(0.19)	(0.17)
Model-based	-0.06	-0.11		
	(0.08)	(0.07)		
abs(QB - QA) x Model-based	0.67^{*}	0.76^{*}		
	(0.30)	(0.29)		
w			-0.09	$-0.16^{\dagger}$
			(0.11)	(0.10)
abs(QB - QA) x w			$0.77^{\dagger}$	0.91^{*}
			(0.40)	(0.38)

$^{*}p < 0.05$, $^{\dagger}p < 0.1$

Supplementary Table 3. Coefficients from logit regression models (P(look at higher Q first) ~ abs(QB – QA) x Model-based) with standard errors clustered at the subject level (the mixed-effects model did not converge in the continuous *w* case, so we used this more conservative method instead). Columns 1 and 2 report the results that use a group dummy, columns 3 and 4 report the results with a continuous *w* per subject. Columns 2 (reported in the main text) and 4 display the results without one subject that had an individual coefficient for abs(QB-QA) that was an extreme outlier (~100 times higher than the rest of the subjects).

P (B chosen)	Group comparison		Continuous w	
	Two or more gazes	Exactly two gazes	Two or more gazes	Exactly two gazes
Intercept	-2.72**	-0.26	-2.67**	-1.54**
	(0.28)	(0.40)	(0.29)	(0.58)
$EV_B - EV_A$	0.06**	0.06*	0.06**	0.06*
	(0.02)	(0.02)	(0.02)	(0.02)
B chosen at $t - 1$	2.15**	2.38**	2.17**	2.38**
	(0.28)	(0.32)	(0.28)	(0.32)
First gaze on B	1.29**		1.29**	1.27†
	(0.21)		(0.20)	(0.73)
Last gaze on B	2.08**	-1.36*	2.07**	
	(0.34)	(0.69)	(0.36)	
Last gaze dwell time	-0.40**	-0.83**	-0.39**	-0.80**
	(0.08)	(0.15)	(0.08)	(0.15)
Model-based	-0.56†	-1.42**		
	(0.33)	(0.54)		
Last gaze time × Last gaze on B	0.86**	1.62**	0.84**	1.57**
	(0.12)	(0.25)	(0.13)	(0.26)
Model-based × Last gaze on B	1.01*	2.70**		
	(0.48)	(0.99)		
Model-based × Last gaze time	0.17	0.39*		
	(0.11)	(0.19)		
Model-based × Last gaze on B × Last gaze time	-0.37*	-0.88**		
	(0.16)	(0.33)		
w			-0.81†	-1.81*
			(0.43)	(0.73)
w × Last gaze on B			1.24†	3.14*
			(0.67)	(1.36)
w × Last gaze time			0.17	0.43†

			(0.15)	(0.26)
w × Last gaze on B × Last gaze time			-0.40	-1.00*
			(0.23)	(0.45)
AIC	2192.82	1423.43	2193.75	1438.30
BIC	2432.12	1599.90	2433.06	1654.62
Log Likelihood	-1057.41	-680.72	-1057.88	-681.15
Num. obs.	3415	2192	3415	2192
Num. groups: id	43	42	43	42

**p < 0.01, *p < 0.05, †p < 0.1

Supplementary Table 4. Replication of Table 1 of the main text using an alternative specification of symbol values, which are calculated as expected values of the accumulated reward for each symbol, using the true action-state transition probabilities (0.7 and 0.3).

P (B chosen)	All data	Model-based	Model-free
Intercept	-0.77^{**}	-1.04^{***}	-1.80^{***}
	(0.29)	(0.31)	(0.40)
QB – QA	3.97^{***}	3.20^{***}	4.84^{***}
	(0.39)	(0.41)	(0.65)
First gaze on B	0.98^{***}	1.14^{***}	0.80^{**}
	(0.20)	(0.30)	(0.27)
Last gaze on B	1.60^{***}	1.87^{***}	2.84^{***}
	(0.45)	(0.46)	(0.50)
Last gaze dwell time	-0.49^{***}	-0.49^{***}	-0.27^{**}
	(0.09)	(0.10)	(0.10)
Gaze time × Last gaze on B	0.82^{***}	0.82^{***}	0.56^{***}
	(0.14)	(0.15)	(0.16)
Model-based	-1.36^{***}		
	(0.41)		
Model-based × Last gaze on B	1.59^{*}		
	(0.66)		
Model-based × Gaze time	0.24^{*}		
	(0.11)		
Model-based × Last gaze on B × Gaze time	-0.30		
	(0.18)		
AIC	2166.96	1319.32	872.16
BIC	2359.43	1470.76	1018.30
Log Likelihood	-1052.48	-632.66	-409.08
Num. obs.	3673	2016	1657
Num. groups: id	43	22	21

$^{***}p < 0.001$, $^{**}p < 0.01$, $^{*}p < 0.05$. Standard errors in parentheses.

Supplementary Table 5. Fixed effects coefficient estimates of **second-stage** choice regressions in condition 1 of the experiment (symbols are arbitrarily labeled A and B) using mixed effects logistic models, trials with two or more symbols attended. Model-based behavior is more affected by the gaze site, while model-free behavior is more influenced by gaze durations.

	All data	Model-free	Model-based
Intercept	-1.37^{***}	-1.32^{**}	-2.35^{***}
	(0.41)	(0.44)	(0.54)
QB – QA	0.12	0.13	-0.02
	(0.43)	(0.55)	(0.89)
First gaze on B	0.97^{***}	1.26^{***}	0.65^{*}
	(0.21)	(0.30)	(0.32)
Last gaze on B	2.04^{***}	**1.88****	**3.47*****
	(0.52)	(0.60)	(0.59)
Last gaze dwell time	-0.38^{***}	-0.48^{***}	-0.20
	(0.09)	(0.11)	(0.11)
Gaze time × Last gaze on B	0.69^{***}	0.82^{***}	0.47^{**}
	(0.14)	(0.18)	(0.16)
Model-based	-1.18		
	(0.64)		
Model-based × Last gaze on B	**1.46**†		
	(0.80)		
Model-based × Gaze time	0.16		
	(0.12)		
Model-based × Last gaze on B × Gaze time	-0.17		
	(0.19)		
AIC	1847.81	1093.62	784.20
BIC	2027.57	1234.31	918.89
Log Likelihood	-892.90	-519.81	-365.10
Num. obs.	2438	1354	1084
Num. groups: id	42	22	20

$^{***}p < 0.001$, $^{**}p < 0.01$, $^{*}p < 0.05$, $\dagger p < 0.1$. Standard errors in parentheses.

Supplementary Table 6. The same analysis as in Supplementary Table 5, with only ***common*** transition trials included. Fixed effects coefficient estimates of **second-stage** choice regressions in condition 1 of the experiment (symbols are arbitrarily labeled A and B) using mixed effects logistic models, trials with two or more symbols attended, only common transition trials included. Model-based subjects are more influenced by the last gaze location (in bold).

	**All data**	**Model-free**	**Model-based**
Intercept	-1.21**	-1.47**	-1.33**
	(0.37)	(0.53)	(0.42)
QB – QA	0.42	0.78	-0.83
	(0.36)	(0.51)	(0.78)
First gaze on B	0.67*	1.07*	0.34
	(0.26)	(0.52)	(0.35)
Last gaze on B	2.75***	**2.88******	**2.95******
	(0.51)	**(0.64)**	**(0.57)**
Last gaze dwell time	-0.31***	-0.43**	-0.20*
	(0.09)	(0.14)	(0.09)
Gaze time × Last gaze on B	0.44***	0.64***	0.33**
	(0.13)	(0.19)	(0.13)
Model-based	-0.39		
	(0.45)		
Model-based × Last gaze on B	**0.25**		
	**(0.68)**		
Model-based × Gaze time	**0.06**		
	**(0.12)**		
Model-based × Last gaze on B × Gaze time	**-0.04**		
	**(0.16)**		
AIC	975.06	518.31	483.79
BIC	1133.74	639.68	601.27
Log Likelihood	-456.53	-232.15	-214.90
Num. obs.	1235	662	573
Num. groups: id	43	22	21

***$p < 0.001$, **$p < 0.01$, *$p < 0.05$. Standard errors in parentheses.

Supplementary Table 7. The same analysis as in Supplementary Table 3, with only ***rare*** transition trials included. Fixed effects coefficient estimates of **second-stage** choice regressions in condition 1 of the experiment (symbols are arbitrarily labeled A and B) using mixed effects logistic models, trials with two or more symbols attended, only "common" transition trials included. We observe no significant difference in coefficients for the two groups (in bold).

	All data	Model-based	Model-free
Intercept	-2.39***	-2.56***	-2.90***
	(0.31)	(0.40)	(0.41)
QB – QA	3.60***	3.05***	5.30***
	(0.44)	(0.48)	(1.10)
B chosen at t-1	1.61***	1.66***	1.51**
	(0.29)	(0.37)	(0.49)
First gaze on B	1.31***	1.67***	0.93***
	(0.23)	(0.39)	(0.27)
Last gaze on B	2.04***	2.17***	3.25***
	(0.38)	(0.44)	(0.40)
Last gaze dwell time	-0.43***	-0.49***	-0.16*
	(0.10)	(0.11)	(0.07)
Gaze time × Last gaze on B	0.90***	1.00***	0.42***
	(0.15)	(0.20)	(0.12)
Model-based	-0.67		
	(0.36)		
Model-based × Last gaze on B	1.29*		
	(0.55)		
Model-based × Gaze time	0.22		
	(0.13)		
Model-based × Last gaze on B × Gaze time	-0.42*		
	(0.20)		
AIC	1771.60	985.82	819.62
BIC	2006.31	1177.12	1000.27
Log Likelihood	-846.80	-457.91	-374.81
Num. obs.	3036	1747	1289
Num. groups: id	30	16	14

***p < 0.001, **p < 0.01, *p < 0.05. Standard errors in parentheses.

Supplementary Table 8. Table 1 excluding the 13 subjects whose zero-gaze-trial rate was above average.

Supplementary Note 1: Exploratory aDDM analyses

Following Krajbich et al. (2010), we fit an attentional drift-diffusion model (aDDM) that assumes a drift diffusion process that evolves over time as a random walk that starts at 0 and reaches a barrier at -1 or +1. If the subject is looking at symbol A, the decision variable changes with a constant drift rate equal to $d(Q_A - \theta Q_B) + \varepsilon_t$, where d is a scale parameter, Q_A and Q_B are Q-values estimated using the hybrid-learning computational model, θ (from 0, reflecting full gaze bias, to 1, the regular DDM case) is a parameter that reflects the bias towards the item currently looked at, and ε_t is normally distributed noise with mean 0 and standard deviation σ. If the subject is looking at symbol B, the drift rate is equal to $d(Q_B - \theta Q_A) + \varepsilon_t$. The model assumes that the first gaze goes randomly to one of the symbols with probability p estimated from the data, and then gazes alternate between the two symbols until one of the barriers is reached, and that every trial has fixed non-decision time t_{er}.

First, to fit the model, following the standard DDM approach (Ratcliff and McKoon, 2008), we calculated the empirical distribution of response times (RTs) binned into 5 quintiles (0.1,0.3,0.5,0.7,0.9) and 11 choice difficulty bins (QA – QB ranging from -1 to +1 by 0.2) for the pooled subject data and fit it to the simulated aDDM RT-difficulty distributions produced by 5000 randomly drawn sets of parameters $(d, \sigma, \theta, t_{er})$ using the chi-square test (minimizing the χ_2 statistic). These fits provided expected fits to the RT distributions, but did not match the choice probabilities (especially for the model-based group) and largely missed the key trends in the eye-tracking data (Supplementary Figures 1 and 2).

Next, we relied on previous work that showed that θ typically takes on a value of 0.3 across several choice domains. Using this value, we adjusted the other model parameters (d and σ) to achieve the best fit to the model-free data. We identified a set of parameters that provided a substantially improved fit to the choice and eye-tracking data without much of a detriment to the RT fits (see Supplementary Figure 3). The model under-estimates the overall probability that the last gaze is to the chosen item (by approximately 10%), which can be due to the fact that these data do contain some visual search trials.

Finally, we performed a grid parameter search based on the model-free fits to fit the model-based subjects' data. By reducing θ to zero, drastically increasing the drift rate d, and reducing noise we were able to mimic the visual search process by producing a strong bias towards choosing the last-seen item (Supplementary Figure 4). But this adjustment reduces the sensitivity of the choices to the Q-value difference, and the produced RTs are significantly faster (~200ms) than the data. This indicates the inability of the aDDM to simultaneously capture the choice accuracies and short RTs at the same time as the significant bias to choose the last-seen item and the many single-gaze trials, displayed by the model-based subjects' data.
